# Gut microbiome impact on systemic therapy outcomes in metastatic renal cell carcinoma: a systematic review

**DOI:** 10.1007/s00345-026-06386-1

**Published:** 2026-04-12

**Authors:** Filippo Gavi, Martina Bracco, Nicoletta Testori, Francesco Rossi, Daniele Fettucciari, Enrico Panio, Simone Assumma, Pierluigi Russo, Carlo Gandi, Nazario Foschi, Mauro Ragonese, Filippo Turri, Riccardo Bientinesi, Giuseppe Palermo, Chiara Ciccarese, Roberto Iacovelli, Paul T. Kröner, Antonio Gasbarrini, Maria Chiara Sighinolfi, Bernardo Rocco

**Affiliations:** 1 Department Of Urology, Fondazione Policlinico Universitario A. Gemelli IRCCS, Roma, Italy; 2 Department Of Oncology, Fondazione Policlinico Universitario A. Gemelli IRCCS, Roma, Italy; 3 Department of Gastroenterology, Riverside Health System, Newport News, VA USA; 4 CEMAD Digestive Disease Center, Fondazione Policlinico Universitario Agostino Gemelli IRCCS, Università Cattolica del Sacro Cuore, Roma, Italy

**Keywords:** Microbiome, Probiotics, Immune therapy, Kidney cancer

## Abstract

**Background and objective:**

Metastatic renal cell carcinoma (mRCC) treatment with immune checkpoint inhibitors (ICIs) and vascular endothelial growth factor–tyrosine kinase inhibitors (VEGF-TKIs) yields durable benefit in a subset of patients, yet primary resistance remains frequent. Emerging data implicate the gut microbiome as a determining factor of systemic therapy efficacy. This review systematically evaluates clinical evidence on antibiotics (ATBs), probiotics, dietary interventions, and fecal microbiota transplantation (FMT) in modulating the gut microbiome to influence outcomes in adult patients with mRCC.

**Methods:**

Eligible studies included adult (≥ 18 year) RCC cohorts receiving microbiome interventions versus standard care, reporting objective response rate (ORR), progression-free survival (PFS), overall survival (OS), or immune-related adverse events (irAEs).

**Key findings and limitations:**

Six studies (*N* = 4738) met criteria: three retrospective cohorts linking peri-ICI ATB exposure to inferior ORR (12.9–34.8%), shorter PFS (hazard ratios [HR] 1.96–3.10), and OS (HR 3.5); one prospective RCT (*n* = 20) demonstrating engraftment of *Bifidobacterium animalis* yogurt during VEGF-TKI therapy with enrichment of *Akkermansia muciniphila* and trends to longer PFS; and one phase II RCT abstract (*n* = 50) showing FMT from an ICI responder improved 1-year PFS (66.7% vs. 35.0%, *p* = 0.036) and ORR (54% vs. 28%) in pembrolizumab + axitinib recipients.

**Conclusions and clinical implications:**

ATB-induced dysbiosis compromises ICI efficacy in mRCC; probiotics and FMT exhibit promise to augment immunotherapy and targeted therapy. Prospective, biomarker-driven RCTs with standardized microbiome assays are needed before routine clinical implementation.

**Supplementary Information:**

The online version contains supplementary material available at 10.1007/s00345-026-06386-1.

## Introduction

Renal cell carcinoma (RCC) comprises 3–5% of adult malignancies, with ~ 79,000 new cases and 14,000 deaths in Europe in 2023 [1]. For mRCC, VEGF-TKIs and ICIs targeting PD-1/PD-L1 and CTLA-4 have reshaped survival [2–6]. Nonetheless, 30–40% of patients exhibit primary resistance to ICIs, and predictive biomarkers remain suboptimal [7, 8].

The gut microbiome is increasingly recognized as a regulator of systemic immunity and response to cancer therapy [9–11]. Preclinical models demonstrate that commensal taxa, particularly *Akkermansia muciniphila* and *Bacteroides fragilis*, enhance ICI efficacy through dendritic cell activation and CD8⁺ T-cell infiltration [12–14], whereas ATB-induced dysbiosis abrogates these effects [15, 16]. Retrospective clinical analyses correlate recent ATB exposure with inferior ORR, PFS, and OS following ICI therapy [17, 18].

Beyond ICIs, the microbiome may also influence VEGF-TKI responses [19–21]. Early-phase studies suggest that probiotics (e.g., *Bifidobacterium*) and dietary prebiotics engraft in the gut and modulate host metabolism and toxicity [19]. FMT from ICI responders restores antitumor immunity in murine RCC models and is under preliminary clinical investigation [22, 23].

Given the emerging “gut–kidney–immune axis,” we performed a systematic review to evaluate clinical evidence on microbiome modulation in mRCC patients receiving ICIs or VEGF-TKIs, with the aim of guiding future translational research and practice.

## Evidence acquisition

### Search strategy

**Fig. 1:**
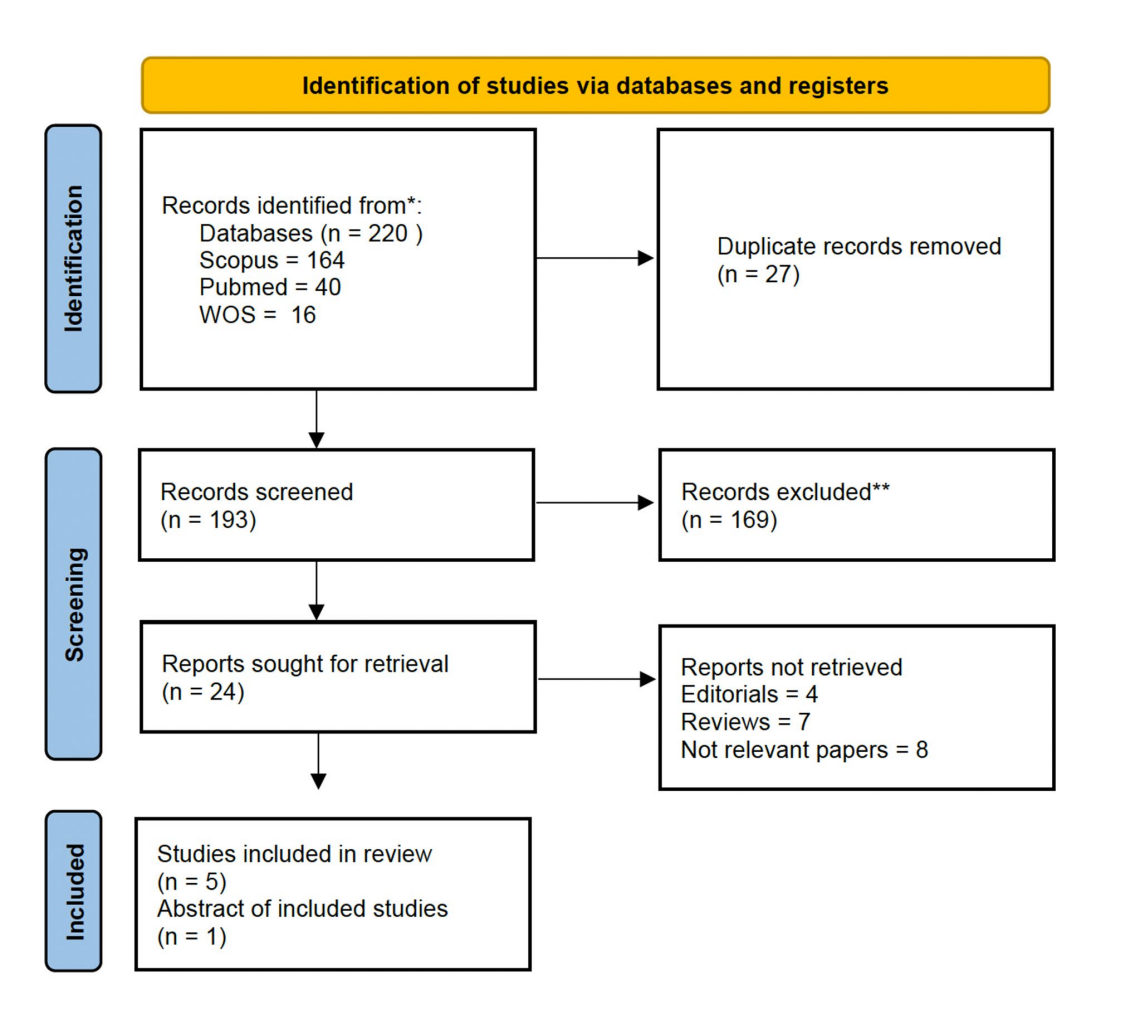
PRISMA flowchart of studies included in the systematic review. Notes: *PRISMA*: Preferred Reporting Items for Systematic Reviews and Meta-analysis; *WOS*: Web of Science

This systematic review was conducted in accordance with the Preferred Reporting Items for Systematic Reviews and Meta-analyses (PRISMA) guidelines. The current protocol was registered in the International Prospective Register of Ongoing Systematic Reviews (PROSPERO) under registration number CRD42025637587. A systematic literature search was performed until January 2025 across the following databases: PubMed, Web of Science, and Scopus. Additionally, the reference lists of eligible articles were hand-searched to identify further relevant studies, including conference abstracts. The searches were not limited by language. Inclusion criteria were: studies involving adult patients (≥ 18 years) with a diagnosis of renal cell carcinoma (RCC), receiving any form of gut microbiome modulation, including but not limited to probiotics, prebiotics, antibiotics, dietary interventions, or fecal microbiota transplantation with or without a comparator group consisting of patients receiving standard oncologic care without targeted microbiome modulation; reporting on clinical outcomes such as treatment response, progression-free survival, overall survival, immune-related adverse events, or quality of life. Exclusion criteria were: (1) non-comparative studies without a control or comparator group; (2) studies not specifying RCC as the cancer type or combining RCC with other malignancies without stratified results; (3) studies conducted in pediatric populations (< 18 years); (4) preclinical studies using animal models or in vitro experiments; and (5) non-original publications, including reviews, editorials, commentaries, or case reports. After duplicate removal, two reviewers (F.G. and M.B.) independently screened the studies to identify the eligible ones. Discrepancies were resolved through discussion and with the consultation of a third author (B.R.). The PRISMA flow chart is shown in Fig. 1.

### Quality assessment

Two reviewers (M.B. and N.T.) independently evaluated the methodological quality of all included studies. Discrepancies were resolved by discussion and, when necessary, adjudication by a third reviewer (F.G.). Assessment tools were selected to match each study design: Observational cohort studies were appraised using the Newcastle–Ottawa Scale (NOS). Randomized controlled trials were assessed with the Cochrane Risk of Bias 2 (RoB 2) tool.

## Evidence synthesis

### Risk of bias

The risk of bias in the included studies was evaluated using the Newcastle-Ottawa Scale (NOS) for cohort studies, which assesses three domains: selection (up to 4 stars), comparability (up to 2 stars), and outcome (up to 3 stars), with a total possible score of 9 stars indicating high quality. Detailed results can be found in Supplementary Table 1. All three studies, Derosa et al. (2018) [16], Ebrahimi et al. (2024) [21], and Lalani et al. (2019) [17] received a NOS score of 7 out of 9, suggesting moderate to good quality overall. In the selection domain, each study earned stars for representative exposed cohorts, drawing non-exposed cohorts from the same source or trial database, ascertaining exposure (e.g., antibiotic use) from records, and confirming absence of outcomes at baseline. For comparability, Derosa et al. and Ebrahimi et al. adjusted for key confounders in multivariate Cox models, earning one star, while Lalani et al. adjusted for IMDC risk and other factors but lacked a second adjustment, limiting its score. The outcome domain was strong across studies, with RECIST-defined progression-free survival or objective responses, adequate follow-up (at least 12 months), and sufficient completeness, though Lalani et al. noted a median follow-up of 12 months without specifying additional adjustments.

Dizman et al. (2020) [20] showed some concerns overall, mainly due to issues in the randomization process and potential deviations from the intended intervention (D1–D2 rated with “-”), while domains related to missing data, outcome measurement, and selective reporting (D3–D5) were judged at low risk. Porcari et al. (2026) [19] also had an overall judgment of some concerns, with concerns across randomization, deviations from intervention, missing outcome data, and selection of the reported result, but a low risk of bias for outcome measurement. RoB-2 detailed results can be found in Supplementary Fig. 1.

### Baseline characteristics

Our initial search identified 220 studies. After initial screening and full text review, 6 studies involving a total of 4738 patients were included. Experimental cohorts (ATB/FMT/probiotic) = 870 patients and control cohorts (no-ATB/placebo/standard) = 3949 patients. In total, six studies were identified: six peer-reviewed full-text articles. Three employed retrospective cohort designs and three were prospective randomized interventional studies. All six investigated the role of gut microbiota either perturbed by ATBs or modulated via probiotics or FMT in shaping clinical outcomes for mRCC patients treated with systemic therapies, predominantly ICIs and VEGF-TKIs. Characteristics and outcomes of the studies included are reported in Table 1.

**Table 1:** Characteristics and outcomes of the studies included

Study (year)	Design	Patients (mRCC)	Intervention	Comparator	Outcomes	Key Findings
Derosa et al. (2018)	Retrospective cohort	121	ATB within 30 d of ICI start	No ATB	ORR, PFS, OS	ATB: ↑progression (75% vs. 22%); PFS 1.9 vs. 7.4 mo (HR 3.1); OS 17.3 vs. 30.6 mo (HR 3.5)
Lalani et al. (2019)	Retrospective cohort	146	ATB 8 wk before to 4 wk after ICI start	No ATB	ORR, PFS	ATB: lower ORR (12.9% vs. 34.8%); shorter PFS (adjusted HR 1.96)
Derosa et al. (2020)	Prospective study	69	ATB within 60 d of nivolumab	No ATB	ORR, PFS, microbiome	ATB: ORR 9% vs. 28%; ↑Clostridium hathewayi; murine FMT restores ICI efficacy via Akkermansia muciniphila and Bacteroides salyersiae
Dizman et al. (2021)	RCT pilot	20	Bifidobacterium animalis yogurt with VEGF-TKI	No probiotic	ORR, PFS, microbiome	100% B. animalis engraftment; ↑Akkermansia muciniphila, Barnesiella intestinihominis; exploratory PFS benefit
Ebrahimi et al. (2024)	Phase I RCT	30	CBM588 + cabozantinib + nivolumab	cabozantinib + nivolumab	ORR, PFS	No significant change in Bifidobacterium abundance or alpha diversity with CBM588 supplementation; Enrichment of unclassified Ruminococcaceae identified in probiotic arm; Higher objective response rate: 74% vs. 20% with probiotics; Improved 6-month progression-free survival: 84% vs. 60%
Ciccarese et al. (2024)	Phase II RCT abstract	50	FMT + pembrolizumab + axitinib	Placebo + pembrolizumab + axitinib	1-yr PFS, ORR	FMT: 1-yr PFS 66.7% vs. 35.0% (*p* = 0.036); ORR 54% vs. 28%; no ↑toxicity

*ATB*: antibiotic;* B. animalis*:Bifidobacterium animalis, *C. hathewayi *: Clostridium hathewayi, *FMT*: fecal microbiota transplantation, *HR* :hazard ratio, *ICI*:immune checkpoint inhibitor, mo:months, *mRCC* :metastatic renal cell carcinoma, *NR*:non-responder,*ORR*: objective response rate, *OS*: overall survival, *PFS*:progression-free survival, *RCT*: randomized controlled trial, *VEGF-TKI*: vascular endothelial growth factor–tyrosine kinase inhibitor

### Antibiotic-associated dysbiosis and ICI efficacy

Derosa et al. (2018) [16] performed a retrospective analysis of 121 advanced RCC patients receiving anti–PD-(L)1 therapy, comparing those who had received systemic ATBs within 30 days of ICI initiation (*n* = 16) against ATB-naïve patients (*n* = 105). ATB exposure was significantly associated with primary progressive disease (75% vs. 22%, *p* < 0.01), shorter median progression-free survival (PFS: 1.9 vs. 7.4 mo; hazard ratio [HR] 3.1, *p* < 0.01), and reduced overall survival (OS: 17.3 vs. 30.6 mo; HR 3.5, *p* = 0.03). Lalani et al. [17] extended these observations in a second retrospective mRCC cohort (*n* = 146), defining ATB use from 8 weeks before to 4 weeks after ICI start. Antibiotic users (*n* = 31) had a lower objective response rate (ORR: 12.9% vs. 34.8%, *p* = 0.026) and shorter PFS (adjusted HR 1.96, *p* = 0.007) compared to non-users, even after controlling for International Metastatic RCC Database Consortium risk factors. Both studies thus consistently demonstrate that recent ATB use compromises ICI efficacy in mRCC.

### Microbiota signatures predictive of ICI response

Derosa et al. (2020) [18] conducted a translational substudy of the NIVOREN-GETUG AFU 26 phase II trial, prospectively collecting fecal samples from 69 mRCC patients treated with nivolumab. Antibiotic‐exposed patients (16%) exhibited markedly lower ORR (9% vs. 28%, *p* < 0.03) and distinct microbial shifts, including overrepresentation of *Clostridium hathewayi*. In parallel, murine models receiving FMT from clinical responders restored ICI sensitivity, pinpointing *Akkermansia muciniphila* and *Bacteroides salyersiae* as key commensals linked to antitumor immunity. These findings bridge clinical correlations with mechanistic proof-of-concept, underscoring specific bacterial taxa as modulators of ICI response.

### Probiotic supplementation during VEGF-TKI therapy

Dizman et al. [20] reported the first prospective, randomized pilot trial assessing whether a *Bifidobacterium animalis*–enriched yogurt could favorably alter the gut microbiome and clinical outcomes in mRCC patients receiving standard first- or subsequent-line VEGF-TKIs (*n* = 20). Although the small sample precluded statistical differences in clinical benefit rate (70% probiotic vs. 80% control, *p* = 0.61), probiotic-supplemented patients uniformly demonstrated increased fecal *B. animalis* levels and enrichment of other potentially beneficial taxa, notably *Akkermansia muciniphila* and *Barnesiella intestinihominis*, which correlated with clinical benefit across timepoints. This study provides proof that dietary probiotics can engraft and modulate the microbiome of patients with mRCC during targeted therapy. Ebrahimi et al. [21] reported data on the use of a probiotic (CBM588) during VEGF TKI therapy. Specifically with cabozantinib plus nivolumab in metastatic renal cell carcinoma, CBM588 did not significantly alter gut Bifidobacterium abundance or microbial diversity but yielded higher response rates (74% vs. 20%) and improved 6‑month PFS (84% vs. 60%) without additional toxicity. The trial identified enrichment of *Ruminococcaceae* species and vitamin K2 biosynthesis pathways associated with better immune response and clinical benefit. These findings suggest a potential synergistic immunomodulatory role of probiotics in enhancing VEGF TKI plus PD‑1 inhibition efficacy, warranting larger confirmatory studies.

### FMT to enhance ICI plus VEGF-TKI combinations

Porcari et al. [19] published the TACITO phase II RCT, randomizing 50 treatment-naïve mRCC patients receiving pembrolizumab plus axitinib to receive either FMT from a long-term ICI responder or placebo. Results in 45 evaluable patients showed a significantly higher median PFS with FMT (14 vs. 9 mo) and ORR (52% vs. 32%), and no increase in grade ≥ 3 adverse events. These data suggest that targeted microbial reconstitution may potentiate combined immuno-angiogenic regimens in mRCC.

## Discussion

Our review highlights that gut microbiome composition is a critical determinant of systemic therapy outcomes in patients with mRCC. Three retrospective cohorts link peri-ICI ATB exposure with inferior ORR, PFS, and OS, indicating that antibiotic-induced dysbiosis undermines ICI efficacy [17, 18, 20]. Prospective microbiome profiling identifies key taxonomic shifts, depletion of *Akkermansia muciniphila*, and expansion of *Clostridium hathewayi* correlates of resistance. Crucially, murine FMT from clinical responders restores ICI sensitivity, providing mechanistic proof-of-concept [20]. Interventional strategies have begun to translate these insights to the clinic. A randomized pilot trial demonstrated that *Bifidobacterium animalis* yogurt supplementation during VEGF-TKI therapy achieves uniform engraftment and enriches beneficial taxa (including *A. muciniphila* and *Barnesiella intestinihominis*), with trends toward improved PFS and reduced TKI-associated diarrhea [20]. The TACITO phase II RCT abstract further suggests that colonic FMT from a durable ICI responder can double the 1-year PFS rate and improve ORR when combined with pembrolizumab + axitinib, without additional grade ≥ 3 toxicity [19]. The gut microbiota modulates anticancer immunity through metabolite production (SCFAs), maintenance of mucosal integrity, immune priming, and antigen mimicry [12–14]. ATB-induced dysbiosis disrupts these processes by depleting SCFA-producing commensals and facilitating pathobiont expansion, leading to a tolerogenic immune milieu [15, 16]. Preclinical interventions with inulin gels or defined commensal consortia in mouse models have successfully reversed primary ICI resistance, further validating the gut–tumor immune axis [12, 22]. The gut microbiome remains a promising but complex target, influenced by numerous uncontrolled cofactors including diet, regional habits, and prior comorbidities. Comparative evaluation with large, prospective datasets is essential to contextualize these findings.

### Clinical Implications

Antibiotic stewardship is paramount: where possible, avoid broad-spectrum antibiotics within 60 days of initiating ICI therapy, as multiple retrospective analyses have demonstrated that such exposure markedly reduces ORR, shortens PFS (HRs 1.96–3.10) and diminishes OS (HR 3.5) [15–17]. If antimicrobial therapy is indispensable, clinicians should preferentially select narrow-spectrum agents and limit the duration to the shortest effective course. Concurrently, baseline microbiome profiling via stool sequencing may identify patients at heightened risk for ICI resistance, for example those with low abundance of *Akkermansia muciniphila*, enabling preemptive interventions [18]. For patients receiving VEGF-TKIs or ICIs, the use of defined probiotic adjuncts such as *Bifidobacterium animalis* or *A. muciniphila* can reliably engraft these taxa, enrich other immunostimulatory microbes, and has been associated with exploratory improvements in PFS and toxicity profiles [20, 21]. More advanced strategies include establishing standardized FMT programs that utilize stool from rigorously screened, long-term ICI responders; early phase II data suggest that such FMT may double 1-year PFS and substantially increase ORR without added grade ≥ 3 toxicity [24]. Finally, dietary interventions, particularly promoting high-fiber diets and prebiotic supplementation, support the proliferation of SCFA-producing bacteria, which have been shown to bolster antitumor immunity in preclinical models.

### Limitations

Current evidence is limited by retrospective designs, small randomized cohorts, heterogeneous microbiome methodologies, and preliminary abstract data. Confounding factors such as diet, proton pump inhibitors, and steroid use were not consistently controlled. Long-term safety of high-dose probiotics and FMT in immunocompromised patients remains to be established. Standardization of microbiota assessment methods is not feasible yet, the direct comparison between studies remains challenging due to methodological heterogeneity.

### Future directions

Future research must prioritize large-scale, biomarker-driven randomized controlled trials in mRCC that stratify patients by baseline microbiome characteristics such as *Akkermansia muciniphila* abundance and recent antibiotic exposure, to validate microbial signatures as predictive biomarkers and to tailor microbiome-modulating interventions [18]. Equally critical is the standardization of microbiome assay methodologies encompassing stool collection protocols, sequencing depth, and unified bioinformatic pipelines to ensure reproducibility and enable meaningful meta-analyses. Integrative multi‐omics approaches that combine metagenomics, metabolomics, and host immune profiling will be essential to dissect the complex microbial–host immune interactions underpinning therapeutic response and resistance [12, 25]. Complementary dietary and lifestyle intervention trials including high‐fiber regimens and targeted prebiotics should be conducted in randomized settings to assess their synergistic effects with ICIs and VEGF‐TKIs, building on preclinical evidence that inulin gel can overcome primary ICI resistance [12]. Finally, the establishment of safety registries for probiotic and FMT use in oncology is imperative to monitor for potential adverse events, such as bacteremia or horizontal gene transfer, and to inform risk–benefit assessments in immunocompromised patient populations.

## Conclusions

This systematic review underscores the pivotal role of the gut microbiome in shaping systemic therapy efficacy in patients with metastatic RCC. Antibiotic-induced dysbiosis around ICI initiation consistently predicts poorer objective responses and survival, whereas targeted modulation through defined probiotic supplements, dietary strategies, or fecal microbiota transplantation has shown early feasibility and signals of enhanced progression-free survival and response rates. Such interventions appear to amplify antitumor immunity and can even reverse primary ICI resistance in preclinical models. Looking ahead, microbiome modulation holds promise as a low-toxicity adjunct to existing RCC therapies and may pave the way toward truly personalized oncology. However, before routine clinical adoption, key challenges must be addressed: large, biomarker-driven trials to validate predictive microbial signatures; harmonization of stool-collection protocols, sequencing methods, and analytic pipelines; and the design of standardized, GMP-grade microbial consortia or dietary regimens. It is also critical to include diverse patient populations to account for regional and ethnic variability in baseline microbiota. Until robust phase II/III data emerge, the use of microbiome-targeted agents cannot be recommended as standard of care. Nevertheless, participation in rigorously controlled clinical trials particularly those evaluating probiotics, prebiotics, and FMT in combination with ICIs or VEGF-TKIs should be strongly encouraged. Such efforts will accelerate the translation of microbiome science into practice and may ultimately transform the therapeutic landscape for patients with advanced RCC.

## Supplementary Information

Below is the link to the electronic supplementary material.

Supplementary Material 1

## Data Availability

all data are avaiable on Pubmed; Scopus and Web of Science.

## Abbreviations

mRCC: Metastatic renal cell carcinoma
ICIs: Immune checkpoint inhibitors
VEGF-TKIs: Vascular endothelial growth factor–tyrosine kinase inhibitors
ATBs: Antibiotics
FMT: Fecal microbiota transplantation
ORR: objective response rate
PFS: progression-free survival
OS: overall survival
irAEs: immune-related adverse events
RCTs: randomized controlled trials
RCC: Renal cell carcinoma
PRISMA: Preferred Reporting Items for Systematic Reviews and Meta-analyses
