# Supplementary material for: Gut microbiome impact on systemic therapy outcomes in metastatic renal cell carcinoma: a systematic review
Source: World J Urol. 2026 Apr 12;44(1):302. doi: 10.1007/s00345-026-06386-1 (PMC13070972; doi:10.1007/s00345-026-06386-1)
Supplement: Supplementary file 1 — Supplementary Material 1 [file 345_2026_6386_MOESM1_ESM.docx]

**Supplementary Table 1.** Quality assessment

| Newcastle–Ottawa Scale (NOS) | | | | |
| --- | --- | --- | --- | --- |
| Study (year) | Selection (max 4) | Comparability (max 2) | Outcome (max 3) | NOS stars (out of 9) |
| Derosa et al. (2018) | • Representative exposed cohort ★;  • Same source for non-exposed ★;  • Exposure ascertained from records ★;  • Outcome absent at start ★ | • Adjusted for key confounders ★;  • Additional adjustment (multivariate Cox) ☆ | • Outcome by RECIST (objective) ★;  • ≥12 mo follow-up ★;  • Adequate completeness of follow-up ☆ | 7/9 |
| Ebrahimi et al. (2024) | • Representative exposed cohort ★;  • Same source for non-exposed ★;  • Exposure ascertained from records ★;  • Outcome absent at start ★ | • Adjusted for key confounders ★;  • Additional adjustment (multivariate Cox) ☆ | • Outcome by RECIST (objective) ★;  • ≥12 mo follow-up ★;  • Adequate completeness of follow-up ☆ | 7/9 |
| Lalani et al. (2019) | • Representative cohort ★; • Non-exposed from same trial database ★; • Exposure (ATB use) from records ★;  • No outcome at baseline ★ | • Adjusted for IMDC risk & other factors ★;  • No second factor ☆ | • RECIST-defined PFS/OS ★;  • Long follow-up (median >12 mo) ★;  • Follow-up completeness ☆ | 7/9 |
| Derosa et al. (2020) | • Prospective cohort of advanced RCC ★;  • Comparator (non-exposed) same center ★;  • Exposure (microbiota & ATB) correctly measured ★;  • Outcome not pre-existent ★ | • Controlled for prior therapies & confounders ★;  • No additional factor ☆ | • Objective microbiome & clinical outcomes ★;  • Adequate follow-up (data lock Sept 2018) ★;  • Loss-to-follow-up not reported ☆ | 7/9 |


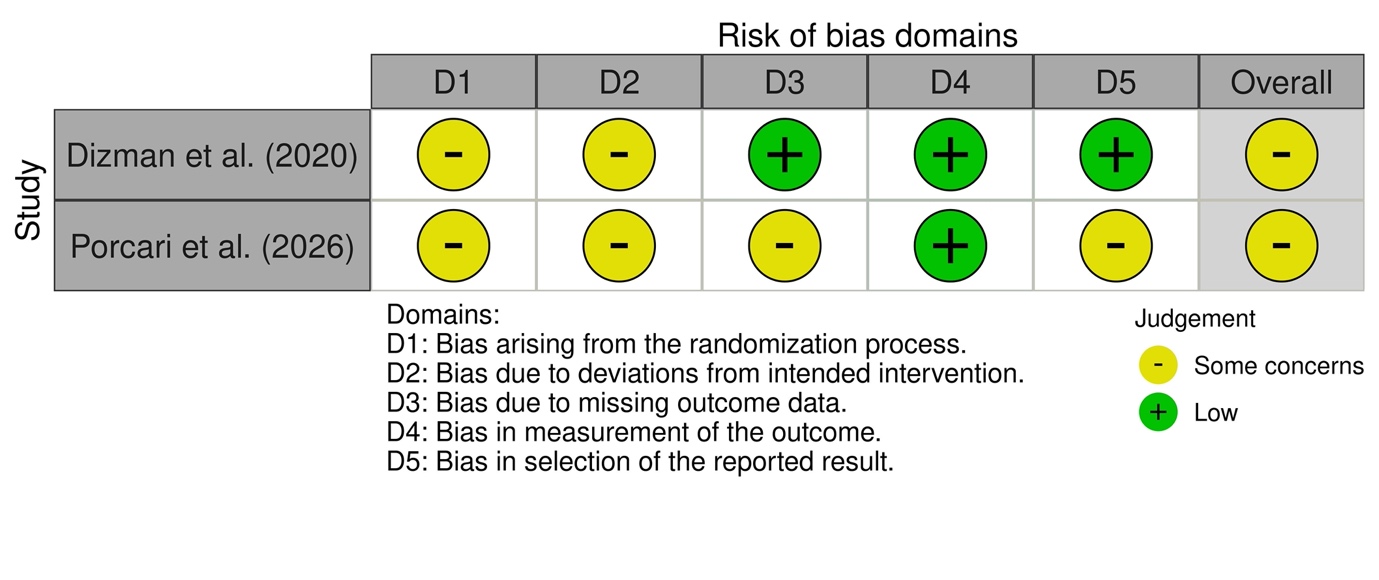
Supplementary Figure 1.
